# Hamstring autografts favour knee extension strength recovery while quadriceps autografts optimise flexion strength recovery: A systematic review of randomised controlled trials

**DOI:** 10.1002/jeo2.70665

**Published:** 2026-02-18

**Authors:** Maximilian Heinz, Jonathan Lettner, Yasemin Begüm Topkarci, Aleksandra Królikowska, Nikolai Ramadanov, Marko Ostojic, Roland Becker, Robert Prill

**Affiliations:** ^1^ Brandenburg Medical School, Center of Orthopedics and Traumatology University Hospital Brandenburg/Havel Brandenburg an der Havel Germany; ^2^ Faculty of Health Science Brandenburg Medical School Brandenburg an der Havel Germany; ^3^ Faculty of Medicine Gazi University Ankara Türkiye; ^4^ Physiotherapy Research Laboratory University Centre of Physiotherapy and Rehabilitation, Faculty of Physiotherapy, Wroclaw Medical University Wroclaw Poland; ^5^ Osteon Orthopedics and Sports Medicine Clinic Mostar Bosnia and Herzegovina; ^6^ Sports Traumatology Division University Hospital “Sisters of Mercy” Zagreb Croatia

**Keywords:** anterior cruciate ligament reconstruction, graft, hamstring graft, muscle strength, outcome, patellar tendon graft, quadriceps graft

## Abstract

**Purpose:**

Quadriceps tendon (QT), hamstring tendon (HT) and patellar tendon (PT) autografts are the most common grafts for anterior cruciate ligament reconstruction (ACLR). However, the specific impact of graft selection on postoperative knee extension and flexion strength remains controversial. This systematic review analysed randomised controlled trials (RCTs) comparing isokinetic muscle strength outcomes following primary ACLR using QT, HT or PT autografts.

**Methods:**

A systematic review was conducted by searching Medline (PubMed), Epistemonikos and ScienceDirect (January 2014–10 May 2025) to identify RCTs that included patients aged ≥16 years undergoing primary, unilateral ACLR using an ipsilateral QT, HT or PT autograft. Studies had to report isokinetic strength outcomes at predefined postoperative intervals (approximately 3, 6, 12 and/or ≥ 24 months) and at one or more angular velocities. The PRISMA guidelines were followed for literature analysis. Two reviewers independently screened, extracted and appraised data using the JBI Checklist for RCTs. A narrative synthesis was performed due to significant clinical and methodological heterogeneity among the included studies.

**Results:**

Of 1509 records screened, 13 RCTs met the criteria, encompassing 5 studies using QT, 13 HT and 4 PT. The synthesis revealed distinct recovery patterns: HT autografts showed more favourable recovery of knee extension strength, particularly between 6 and 12 months postoperatively. Conversely, QT autografts consistently resulted in superior recovery of knee flexor strength across all follow‐up periods. The results for PT autografts were heterogeneous, showing some short‐term advantages but no consistent superiority. Patient‐reported outcomes (PROMs) generally improved in all groups, though patterns varied by instrument and time point.

**Conclusion:**

Current evidence indicates graft‐specific profiles of strength recovery after ACLR, with HT autografts favouring knee extension and QT autografts favouring knee flexion recovery. However, heterogeneity in testing protocols, angular velocities and the reporting of limb symmetry indices limits the strength of these conclusions.

**Level of Evidence:**

Level I.

## Graphical abstract

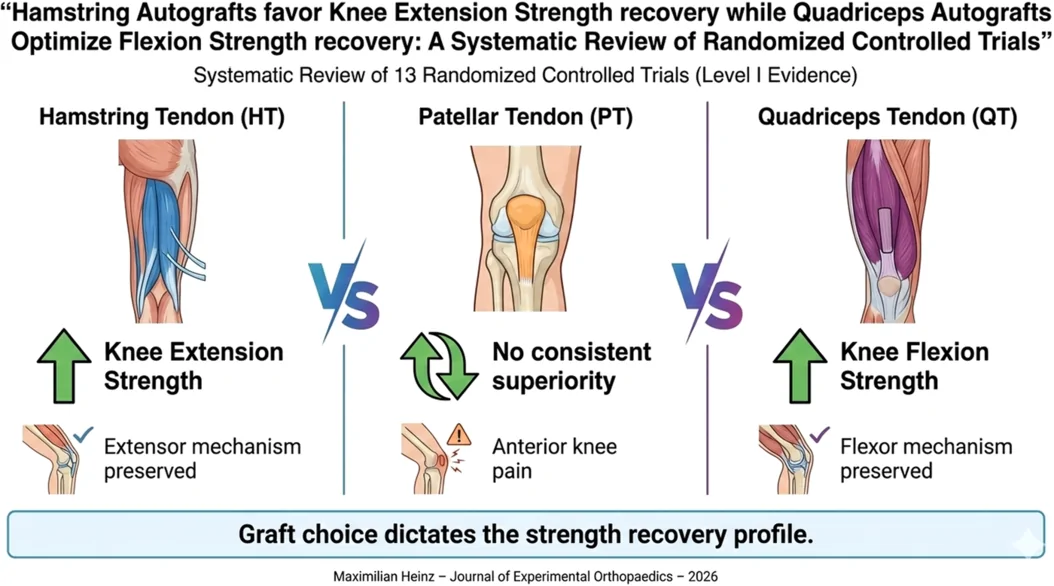

**Abbreviations**
2ST/G: two‐strand semitendinosus/gracilis
4ST: four‐strand semitendinosus
4ST/G: four‐strand semitendinosus/gracilis
Acc.: accelerated
ACL: anterior cruciate ligament
ACLR: anterior cruciate ligament reconstruction
BMI: body mass index
BPTB: bone–patellar tendon‐bone
HT: hamstring tendon
H/Q Ratio: hamstring to quadriceps ratio
IKDC: International Knee Documentation Committee
JBI: Joanna Briggs Institute
KOOS: knee injury and osteoarthritis outcome score
LSI: limb symmetry index
NR: not reported
PRISMA: preferred reporting items for systematic reviews and meta‐analyses
PROMs: patient‐reported outcome measures
PT: patellar tendon
QT: quadriceps tendon
RCT: randomised controlled trial
RTS: return to sport
SD: standard deviation
Std.: standard
ST/G: semitendinosus/gracilis

## INTRODUCTION

Anterior cruciate ligament (ACL) injuries are common and clinically significant knee pathologies, particularly affecting young and physically active individuals. They impose a considerable personal and socioeconomic burden due to both acute impairment and long‐term consequences such as activity limitations and an increased risk of early‐onset osteoarthritis [8, 17, 20]. For patients with functional knee instability who wish to return to sport (RTS), ACL reconstruction (ACLR) is the treatment of choice [5].

Despite generally successful restoration of mechanical stability, ACLR does not consistently result in full functional recovery. A considerable proportion of patients continue to experience persistent quadriceps and hamstring weakness, sometimes lasting years after surgery [9, 21]. These residual deficits are clinically relevant, as quadriceps weakness has been associated with poorer functional outcomes, delayed or incomplete RTS and even a higher risk of secondary ACL injury [19]. Similarly, inadequate hamstring recovery is of particular concern, given the hamstrings' role as dynamic stabilisers counteracting anterior tibial translation. Persistent muscle weakness therefore represents a key modifiable factor linking ACLR to reinjury and suboptimal long‐term outcomes.

In surgical practice, autografts from the quadriceps tendon (QT), hamstring tendon (HT), patellar tendon (PT) and bone–patellar tendon‐bone (BPTB) are the most widely used options for primary ACLR. Each graft type has distinct biomechanical properties, donor‐site morbidities and rehabilitation profiles. However, their specific influence on postoperative strength recovery remains controversial. The available evidence is inconsistent and often limited by methodological heterogeneity, including differences in isokinetic testing procedures, angular velocities, follow‐up time points and rehabilitation regimens [31, 38, 42].

Particular importance has been attributed to the balance between quadriceps and hamstring strength, commonly expressed as the quadriceps‐to‐hamstring (Q/H) ratio. Hamstring strength is equally vital due to its synergistic stabilising function. Acting as dynamic antagonists to the quadriceps, the hamstrings generate a posterior force on the tibia that counterbalances anterior shear stresses and thereby reduces strain on passive stabilisers such as the ACL. This mechanism is relevant in many sporting situations, but especially critical during high‐load movement patterns like deceleration, landing or pivoting, where optimal synergistic and antagonistic muscle activation ensures functional joint stability. By ensuring joint congruency and supporting neuromuscular control, the hamstrings provide a crucial protective buffer that not only prevents injury but also enables efficient and controlled athletic movements [12, 41].

As such, understanding graft‐related differences in extensor and flexor recovery is not only of academic interest but also of direct practical relevance for individualised rehabilitation and return‐to‐sport strategies.

Against this background, the present systematic review aims to synthesise evidence from randomised controlled trials (RCTs) directly comparing QT, HT and PT autografts. It was hypothesised that graft choice differentially affects postoperative isokinetic strength recovery, with greater hamstring deficits after HT and greater quadriceps deficits after QT or PT. The primary focus was on the assessment of postoperative isokinetic strength recovery of both quadriceps and hamstrings, assessed across multiple angular velocities and time points. By clarifying graft‐specific recovery profiles, this review seeks to support evidence‐based graft selection, which may help to optimise rehabilitation protocols and ultimately improve patient outcomes after ACLR.

## MATERIALS AND METHODS

### Protocol and registration

This systematic review followed the Preferred Reporting Items for Systematic Reviews and Meta‐Analyses guidelines (PRISMA) and the author guidelines for conducting systematic reviews and meta‐analyses [26, 29]. Compliance with these guidelines ensures a transparent and structured approach to the conduct and reporting of systematic reviews.

This review strictly adheres to its protocol, which was preregistered in the International Prospective Register of Systematic Reviews (PROSPERO) under the registration number CRD420251055328.

### Literature search

A comprehensive literature search was performed in Medline (PubMed), Epistemonikos and ScienceDirect for studies published from January 2014 to 10 May 2025. The following search terms were used: (“anterior cruciate ligament”[Title/Abstract] OR “acl”[Title/Abstract]) AND (“strength”[Title/Abstract] OR “power”[Title/Abstract] OR “isokinetic”[Title/Abstract] OR “isometric”[Title/Abstract] OR “isoinertial”[Title/Abstract]) AND (“graft”[Title/Abstract] OR “quadricepstendon”[Title/Abstract] OR “bone tendon bone”[Title/Abstract] OR “semitendinosus”[Title/Abstract] OR “gracilis”[Title/Abstract] OR “hamstring”[Title/Abstract] OR “patellar”[Title/Abstract] OR “autograft”[Title/Abstract]). The search query was adapted slightly for each database to maximise retrieval coverage.

### Study selection (inclusion and exclusion)

Studies were selected based on the following eligibility criteria: (1) RCTs comparing two graft options, (2) Both male and female participants aged 16 years or older who had undergone isolated primary ACLR surgery using an ipsilateral autograft (QT, HT, BPTB or PT), (3) postoperative isokinetic peak torque parameters for strength assessment conducted at one or more predefined time points (3, 6, 12 or ≥ 24 months), (4) an accessible full text. For comparison purposes, PT was considered to include both PT and BPTB techniques. HT grafts included semitendinosus/gracilis (ST/G), two‐strand semitendinosus/gracilis (2ST/G), four‐strand semitendinosus (4ST) and four‐strand semitendinosus/gracilis (4ST/G). Articles were considered eligible if at least the abstract was available and the language was accessible to the reviewer team (German, Polish, Turkish or English). When full texts were unavailable, authors were contacted by email; studies were excluded if no response was received within 2 weeks.

### Data extraction and quality assessment

All identified articles were imported into Zotero (Corporation for Digital Scholarship, Vienna, VA, USA) for reference management, and duplicates were removed. Two reviewers (M.H. and Y.B.T.) independently screened the literature using the Rayyan (Qatar Computing Research Institute, Doha, Qatar) web application [25]. The screening process occurred in two stages: an initial review of title and abstract, followed by full‐text evaluation of potentially relevant articles. At each stage, studies were assessed against the predefined eligibility criteria. In cases of disagreement, a third reviewer (J.L.) was consulted to reach a consensus regarding inclusion or exclusion.

Data extraction was performed independently by two researchers (M.H. and Y.B.T.). Discrepancies were resolved by consensus, including a third reviewer (J.L.) and a group‐based discussion with R.P. making the final decision when necessary. The following key data were extracted: first author, year of publication, country of origin and journal. Furthermore, details concerning the type of graft used, along with the specific femoral and tibial fixation methods, were collected. Patient demographics, such as age, body mass index (BMI) and other relevant characteristics, were also recorded. Information on the time required for patients to return to pre‐injury level of sport, the devices used for strength assessment and quantitative data on both extension and flexion strength were extracted. In addition, all reported adverse events and patient‐reported outcome measures (PROMs) were documented.

### Statistical analysis

Due to heterogeneity in outcome definitions and reporting, a formal meta‐analysis was not feasible. Instead, study‐level results were summarised descriptively. For each outcome and time point, the unweighted arithmetic mean of the study‐reported means was calculated, along with the standard deviation across studies (reflecting between‐study variability). No inverse‐variance weighting was applied, and within‐study variances were not pooled.

## RESULTS

### Search results

**Figure 1:**
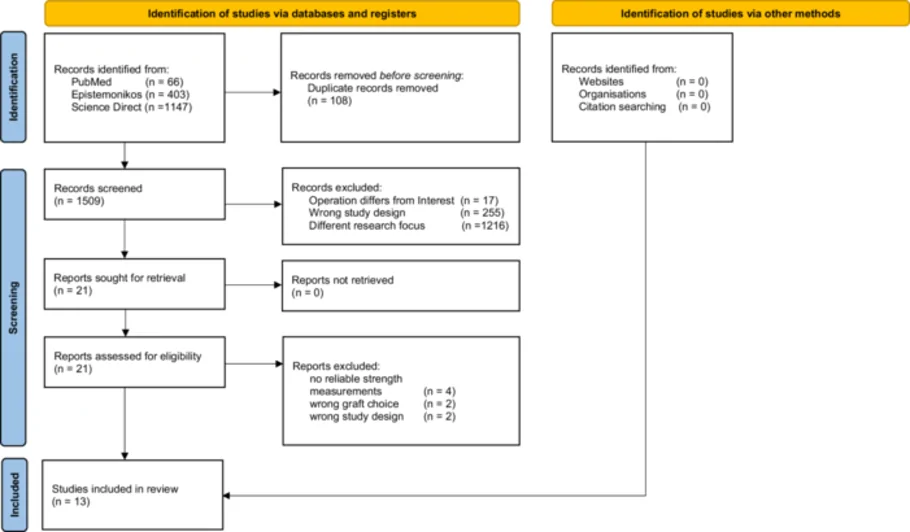
PRISMA flow diagram of study selection.

A total of 1617 studies were identified based on the predefined search criteria. After the removal of 108 duplicates, 1509 unique records remained for screening. During the initial screening phase, 1486 records were excluded because they did not meet the inclusion criteria. The remaining 21 articles were assessed for eligibility via full‐text screening, after which eight studies were excluded for the following reasons: lack of reliable strength measurements (*n* = 4), ineligible graft types (*n* = 2) and non‐RCT study design (*n* = 2). Ultimately, 13 RCTs [1, 4, 6, 14, 18, 19, 22, 23, 28, 32, 34, 36, 37] were included in this systematic review. Across all included studies, 1139 patients were analysed, of whom 734 were male and 405 were female, with a mean age of 29.0 ± 8.7 years and a weighted mean BMI of 24.6 kg/m² in the subset of studies reporting BMI. A detailed overview of the study selection process is presented in the PRISMA flow diagram (Figure 1), and the baseline characteristics of the included populations are summarised in Supplementary Material (Table S1).

### Methodological appraisal of included studies

According to current recommendations, the methodological quality of the included studies was assessed using the JBI Critical Appraisal Checklist for RCTs [2, 30]. Results of the critical appraisal are presented in Table 1. Assessment was conducted independently by two reviewers (M.H. and Y.B.T.), and all extracted data and quality scores were subsequently verified by a third reviewer (J.L.) to resolve any discrepancies.

**Table 1:** Quality assessment of all the included studies using the revised JBI critical appraisal tool for randomised controlled trials.

Critical appraisal according to study design (following JBI critical appraisal tool)													
JBI RCTs	Randomisation	Allocation	Similar groups	Participants blinded	Treatment deliverers blinded	Assessors blinded	Identical treatment in both groups	Outcomes measured same way	Outcomes measured reliable	Follow up complete	Original groups for analysis	Appropriate statistics	Design and modifying appropriate
Arida et al. [1]	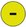	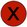	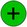	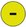	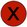	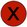	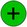	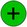	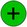	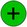	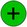	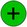	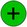
Christiani et al. [4]	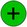	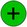	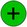	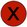	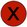	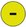	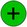	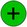	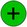	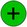	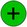	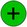	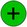
Ebert et al. [6]	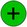	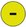	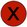	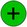	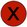	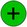	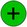	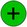	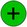	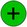	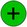	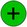	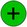
Horstman et al. [14]	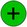	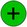	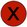	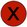	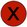	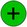	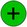	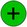	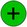	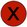	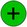	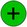	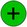
Karimi‐Mobarakeh et al. [18]	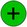	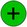	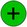	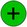	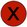	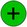	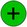	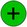	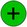	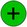	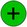	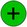	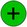
Kouloumentas et al. [19]	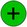	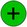	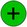	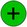	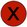	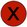	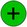	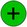	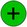	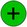	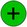	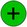	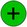
Martin‐Alguacil et al. [22]	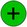	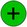	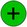	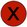	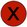	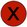	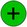	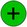	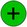	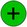	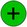	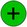	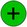
Mo et al. [23]	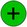	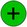	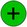	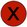	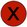	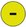	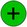	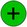	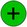	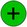	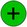	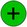	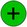
Popovic et al. [28]	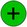	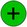	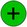	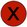	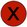	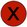	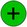	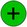	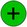	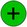	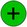	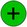	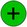
Roger et al. [32]	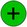	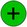	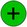	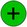	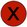	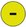	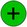	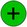	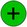	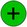	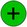	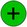	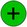
Sasaki et al. [34]	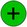	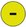	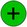	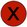	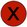	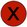	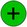	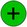	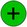	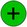	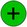	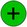	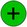
Staghøj et al. [36]	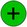	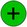	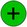	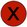	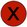	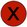	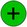	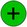	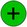	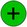	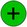	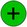	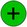
Tang et al. [37]	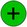	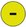	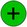	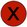	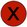	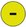	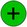	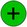	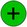	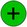	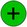	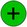	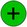

*Note*: (+), low risk of bias; (−), some concerns; (x), high risk of bias.

### Descriptive analysis of the included studies

#### HT versus QT

Ebert et al. [6] compared HT (4ST, *n* = 55) and QT (*n* = 57) autografts in 112 patients undergoing ACLR, with outcomes assessed over 24 months. The HT group showed significantly greater extension strength LSIs at 90°/s at 6, 12 and 24 months, with values of 71.2 ± 16.8, 85.3 ± 13.1 and 92.5 ± 8.2, respectively, compared to 60.9 ± 14.8, 77.3 ± 15.9 and 89.3 ± 10.9 in the QT group. Conversely, the QT group demonstrated superior flexion strength LSIs across the same time points, measuring 95.5 ± 12.8, 94 ± 8 and 101.1 ± 5.1, compared to 87.5 ± 12.1, 93.8 ± 11 and 94 ± 8 in the HT group [6].

Horstmann et al. [14] evaluated 51 patients randomised to four‐strand HT (4HT, *n* = 27) or QT (*n* = 24) autografts. Both graft types yielded comparable improvements in knee stability, with significant gains across all follow‐up intervals. For extension strength at 60°/s (injured–healthy knee, Nm/kg bodyweight), the QT group recorded a decrease of −1.6 ± 0.8 Nm at 3 months, −1 ± 1 Nm at 6 months, −0.6 ± 0.7 Nm at 12 months and −0.3 ± 1.2 Nm at 24 months, compared to −2 ± 1.2 Nm, −1 ± 1.1 Nm, −0.4 ± 0.7 Nm and −0.2 ± 1.2 Nm in the HT group. For flexion strength at 60°/s, the QT group showed −0.5 ± 0.6 Nm, −0.2 ± 0.8 Nm, 0 ± 0.7 Nm and −0.1 ± 1 Nm, while the HT group reported −0.9 ± 0.6 Nm, −0.7 ± 1 Nm, −0.4 ± 0.9 Nm and −0.5 ± 0.8 Nm across the same time points. These results indicated similar recovery profiles between the two techniques [14].

Martin‐Alguacil et al. [22] investigated 51 soccer players randomised to receive either (HT, *n* = 25) or (QT, *n* = 26) grafts, with the hamstring‐to‐quadriceps (H/Q) strength ratio as the primary endpoint. The HT group exhibited greater extensor strength at 3 and 6 months postoperative, measuring 118.4 ± 45.3 Nm versus 85.2 ± 40.1 Nm in the QT group at 3 months, and 139.9 ± 47.9 Nm versus 125.7 ± 40.3 Nm at 6 months. By 12 months, the difference in extension strength had diminished (144.9 ± 51.4 Nm in the HT group vs. 139.5 ± 47.3 Nm in the QT group).

In contrast, flexion strength consistently favoured the QT group across all time points, showing significant differences. At 60°/s, the QT group achieved 112.3 ± 30.9 Nm compared to 104.3 ± 36.1 Nm in the HT group at 3 months, and 147.4 ± 37.8 Nm versus 131.3 ± 40.4 Nm at 12 months. These findings resulted in a superior H/Q ratio for the QT group at 12 months, although both graft types demonstrated comparable functional outcomes after 24 months [22].

Sinding et al. [36] evaluated 85 patients randomised to HT (4ST/G, *n* = 43) or QT (*n* = 42) autografts. At 12 months, the QT group demonstrated significantly weaker extension strength (84.1 ± 12.7 at 60°/s and 83.4 ± 13.4 at 180°/s) compared to 90.2 ± 11.2 and 88.1 ± 10.2 in the HT group. Conversely, the HT group showed significantly lower flexion strength, with LSIs of 85.7 ± 17 at 60°/s and 83.4 ± 17.2 at 180°/s, compared to 92.3 ± 19.2 and 100.2 ± 14.9 in the QT group. No significant differences were observed in SLH or patient‐reported outcomes [36].

Tang et al. [37] compared HT (4ST/G, *n* = 16) and QT (*n* = 17) grafts in 33 patients over 24 months. Both groups achieved comparable PROMs, indicating similar subjective recovery and knee function. However, objective isokinetic testing revealed distinct strength profiles between graft types. At 60°/s, the HT group demonstrated significantly greater knee extension strength (145.06 ± 37.37 Nm) compared to the QT group (119.76 ± 41.99 Nm). In contrast, flexion strength was slightly higher in the QT group (111.88 ± 29.33 Nm) than in the HT group (105.75 ± 22.89 Nm). These results suggest that although functional outcomes were equivalent between groups, the HT graft was associated with better extensor strength recovery, whereas the QT graft provided a modest advantage in flexor strength [37].

#### Quadruple semitendinosus (ST) versus single semitendinosus and gracilis tendon (ST/G)

Karimi‐Mobarakeh et al. [18] investigated whether harvesting the gracilis tendon was necessary in ACLR. In their double‐blind randomised trial, 119 patients were allocated to either a four‐strand ST (4ST, *n* = 58) or semitendinosus plus gracilis (ST/G, *n* = 61). After 12 months, maximal extension strength was 71.8 ± 10.5 Nm for the 4ST group and 73.7 ± 11.8 Nm for the ST/G group, while flexion strength measured 68.3 ± 9.1 Nm and 65.5 ± 8.6 Nm, respectively. No significant differences were observed regarding surgical complications, PROMs or adverse events, leading the authors to conclude that gracilis harvesting may not be necessary [18].

Kouloumentas et al. [19] compared an ‘all‐inside’ ACLR technique using a quadrupled ST (4ST, *n* = 45) to a conventional semitendinosus/gracilis (4ST/G, *n* = 45) graft. Over 2 years, both groups showed similar PROMs and anterior tibial translation, but the all‐inside group demonstrated significantly superior isokinetic flexor strength at 180°/s, with an LSI of 100 ± 10 compared to 90 ± 10 in the conventional group. Time to peak and total work also favoured the all‐inside technique (90 ± 10 vs. 80 ± 10 for both measures). Additionally, the isometric flexor/extensor ratio at 90°/s was significantly higher in the all‐inside group (110 ± 30 vs. 80 ± 20). These findings suggest that the all‐inside technique may better preserve flexor strength [19].

Roger et al. [32] examined 60 patients undergoing ACLR, comparing a 4ST (*n* = 33) graft with cortical fixation to an ST/G (*n* = 27) graft with traditional fixation. Both techniques yielded favourable clinical outcomes, but the 4ST group demonstrated significantly higher flexion strength LSIs at both 60°/s (93.9 ± 12.1 vs. 84.7 ± 13.9) and 180°/s (97.7 ± 11.2 vs. 85.5 ± 14.8). Extension strength did not differ significantly. These results support the all‐inside technique as a viable alternative with superior hamstring strength recovery [32].

Mo et al. [23] compared an all‐inside 4ST (*n* = 48) with a traditional 4ST/G (*n* = 49) graft in 97 patients. No significant differences were observed between groups in PROMs, hamstring strength or knee laxity over 2 years. Extension LSI values at 90°/s after 9 and 12 months were 74.9 ± 16.7 and 87.4 ± 16.5 for the 4ST group, compared to 78.3 ± 24.7 and 88.0 ± 10.4 for the 4ST/G group. For flexion, LSIs were 85.3 ± 15.4 and 88.7 ± 21.3 in the 4ST group, compared to 79.9 ± 2.9 and 86.0 ± 18.6 in the 4ST/G group. These findings indicate that comparable outcomes were observed between the two techniques [23].

#### HT versus BPTB

In a prospective randomised study, Arida et al. [1] investigated 60 amateur athletes undergoing ACLR, comparing four‐strand HT (4HT, *n* = 30) with BPTB (*n* = 30) grafts. The results indicated that the BPTB group demonstrated significantly higher peak torque values during flexion at both 60°/s and 180°/s, at 6 and 12 months postoperative. At 6 months, the BPTB group recorded 98.34 ± 32.07 Nm at 60°/s and 77.86 ± 26.37 Nm at 180°/s, while the HT group reached 79.29 ± 23.07 Nm and 63.13 ± 20.95 Nm, respectively. At 12 months, the BPTB group showed 104.28 ± 31.72 Nm at 60°/s and 85.83 ± 22.36 Nm at 180°/s, compared to 86.17 ± 27.84 Nm and 71.38 ± 24.62 Nm in the HT group. Only minor but statistically significant differences were detected in extension strength, favouring the BPTB graft. Notably, the HT group experienced a significantly higher incidence of ACL rerupture (*n* = 6) compared to the BPTB group (*n* = 1; *p* = 0.044). The authors concluded that both graft types support favourable outcomes in terms of knee function and stability, yet graft choice should be individualised, especially considering the higher rerupture risk associated with HT grafts [1].

Christiani et al. [4] conducted a RCT including 160 patients who were assigned to one of four groups: HT (2ST/G) with standard rehabilitation, HT (2ST/G) with accelerated rehabilitation, BPTB graft with standard rehabilitation or BPTB with accelerated rehabilitation (*n* = 40 in each group). Quadriceps and hamstring strength, along with single‐leg hop (SLH) performance, were followed over 24 months. Extension strength, assessed by the LSI at 90°/s, was consistently lower in the BPTB groups compared to the HT groups at 4, 6, 8 and 12 months. At 4 months, LSI values were 75.8 (HT standard rehabilitation) and 77.4 (HT accelerated rehabilitation), compared to 60 (BPTB standard rehabilitation) and 65.3 (BPTB accelerated rehabilitation). At 6 months, values were 81.6 and 82.9 for the HT groups and 71.4 and 72.2 for the BPTB groups. By 8 months, LSIs had improved to 86.1 and 90.3 for the HT groups and 78.3 and 77.1 for the BPTB groups. At 12 months, the HT groups achieved 90.5 and 92.8, while the BPTB groups showed 85.8 and 85.1. These differences were no longer evident at 24 months. Conversely, flexion strength was significantly lower in the HT groups across all time points [4].

Popovic et al. [28] presented 18‐year follow‐up data from a multicenter trial comparing HT (4ST/G, *n* = 49) with BPTB (*n* = 47) grafts. While functional outcomes were similar, the HT group demonstrated persistent deficits in flexion strength. At 60°/s, maximal flexion strength was 90.6 ± 14.9 Nm in the HT group versus 100.4 ± 14.1 Nm in the BPTB group; at 120°/s, 94.7 ± 14.3 Nm versus 101.4 ± 11 Nm; and at 180°/s, 92.6 ± 11.8 Nm versus 97.8 ± 17.2 Nm. These differences represented a 10.7% reduction in peak flexion torque and 17.2% lower total flexion work in the hamstring group. Extension strength, however, was comparable between grafts [28].

Sasaki et al. [34] compared double‐bundle ACLR using HT (4ST, *n* = 67) with single‐bundle rectangular‐tunnel PT grafts (*n* = 69). At 3 months, extension LSIs were 58 for the HT group and 60 for the PT group; at 6 months, 76 ± 0.16 and 69 ± 0.18; and at 9 months, 84 ± 0.2 and 76 ± 0.76, respectively. For flexion, LSIs were similar: 74 and 76 at 3 months, 88 and 92 at 6 months and 90 and 92 at 9 months. While early postoperative extensor strength deficits were noted in the PT group, differences were no longer significant at 24 months [34].

### Clinical outcomes

Table 2 presents the mean PROMs stratified by graft type. For the Tegner score, the QT demonstrated the highest values at 6 months (6.86 ± 0.85) and 24 months (9.38). However, at 12 months, the PT exhibited a markedly higher score compared to the other grafts (10.97 ± 0.71). Regarding the International Knee Documentation Committee (IKDC) score, the PT achieved the best outcomes at both 6 months (83.28 ± 10.45) and 12 months (89.71 ± 10.48), whereas no values for the PT were reported at 24 months. The Lysholm score displayed a variable pattern: at 6 months, the HT showed the highest score (86.32 ± 6.66), at 12 months the PT led (93.67 ± 3.15), and at 24 months the HT again demonstrated the best result (92.17 ± 5.8). For the knee injury and osteoarthritis outcome score (KOOS), the HT consistently achieved the highest scores across all postoperative time points, with 72.5 ± 5.9 at 6 months, 76.4 ± 5.6 at 12 months and 94.05 ± 3.49 at 24 months. A comprehensive table of all PROMs results is provided in the Supplementary Files (Table S2).

**Table 2:** Overall average of reported PROMs.

	Average score preoperative				Average score after 6 months				Average score after 12 months				Average score after 24 months			
	Tegner ± SD	IKDC ± SD	Lysholm ± SD	KOOS ± SD	Tegner ± SD	IKDC ± SD	Lysholm ± SD	KOOS ± SD	Tegner ± SD	IKDC ± SD	Lysholm ± SD	KOOS ± SD	Tegner ± SD	IKDC ± SD	Lysholm ± SD	KOOS ± SD
**QT**	5.6 ± 0.85	59.85 ± 15.22	66.38 ± 15.82	63.88 ± 14.48	6.86 ± 0.85	71.6 ± 14.8	81.2 ± 12	69 ± 8.6	7.69 ± 0.85	79 ± 15.6	88.3 ± 11.1	73.8 ± 6.8	9.38	88.21 ± 10.14	90.2 ± 8.08	85.59 ± 5.05
**HT**	5.24 ± 0.9	51.38 ± 22.86	57.41 ± 12.8	65.77 ± 11.32	6.4 ± 0.81	77.07 ± 12.38	86.32 ± 6.66	72.5 ± 5.9	6.82 ± 0.68	70.49 ± 10.09	89.61 ± 4.66	76.4 ± 5.6	7.59 ± 0.56	82.42 ± 11.61	92.17 ± 5.8	94.05 ± 3.49
**PT**	6.7 ± 1.6	NR	65	NR	6.14 ± 1.33	83.28 ± 10.45	NR	NR	10.97 ± 0.71	89.71 ± 10.48	93.67 ± 3.15	NR	5.4 ± 1.1	NR	92	NR

Abbreviations: HT, hamstring tendon; IKDC, International Knee Documentation Committee; KOOS, Knee Injury and Osteoarthritis Outcome Score; NR, not reported; PT, patellar tendon; QT, quadriceps tendon; SD, standard deviation.

The strength outcomes in extension indicate a consistent advantage for the HT. At 6 months, LSI values of 78.56 ± 5.6 at 90°/s and 88.1 ± 5.6 at 180°/s were reported. A slightly higher value was observed for the PT at 60°/s; however, this was documented in a single study only [32]. At 12 months, the HT showed the highest LSI values across all angular velocities: 88.1 ± 5.6 at 60°/s, 89.53 ± 4.36 at 90°/s and 88.1 ± 10.2 at 180°/s. At 24 months, the QT demonstrated the highest LSI at 60°/s (119.76 ± 41.99), although this was also reported in only one study [37]. At 90°/s, the HT again achieved the most favourable outcomes (92.4 ± 2.73). Detailed strength values are presented in Table 3.

**Table 3:** Average tendon strength results of the included studies.

6 months 60°/s		6 months 90°/s		6 months 180°/s		12 months 60°/s		12 months 90°/s		12 months 180°/s		24 months 60°/s		24 months 90°/s		24 months 180°/s	
LSI ± SD	Nm ± SD	LSI ± SD	Nm ± SD	LSI ± SD	Nm ± SD	LSI ± SD	Nm ± SD	LSI ± SD	Nm ± SD	LSI ± SD	Nm ± SD	LSI ± SD	Nm ± SD	LSI ± SD	Nm ± SD	LSI ± SD	Nm ± SD
Extension strength averages																	
NR	125.7 ± 40.3	60.9 ± 14.8	NR	84.1 ± 12.7	104 ± 29.1	84.1 ± 12.7	139.5 ± 47.3	77.3 ± 15.9	NR	83.4 ± 13.4	147.4 ± 37.8	119.76 ± 41.9	NR	89.3 ± 10.9	NR	NR	NR
67 ± 16.33	131.64 ± 45.69	78.56 ± 5.6	NR	88.1 ± 5.6	99.32 ± 31.43	88.1 ± 5.6	141.51 ± 50.15	89.53 ± 4.36	72.75 ± 11.15	88.1 ± 10.2	107.24 ± 31.61	94.80 ± 15.65	NR	92.4 ± 2.73	NR	90 ± 10	NR
69 ± 18	125.59 ± 59	71.8	NR	84	93.07 ± 38.4	84	153.17 ± 50.84	85.45	NR	NR	109.9 ± 33.52	89 ± 13	NR	NR	NR	NR	NR
Flexion strength averages																	
NR	136.4 ± 39.8	95.5 ± 12.8	NR	NR	77.86 ± 26.37	92.3 ± 19.2	147.4 ± 37.8	102.9 ± 9.6	NR	100.2 ± 14.9	127.4 ± 34.1	111.88 ± 29.33	NR	101.1 ± 5.1	NR	NR	NR
78.66 ± 8	97.89 ± 32.18	87.06 ± 4.03	NR	NR	86.23 ± 27.57	90.85 ± 8.5	108.73 ± 34.12	89.76 ± 3.66	66.9 ± 39.1	83.4 ± 17.2	91.79 ± 26.97	90.05 ± 5.59	NR	90.7 ± 2.66	NR	95 ± 10	NR
92	98.34 ± 32.07	95.5	NR	NR	77.86 ± 26.37	94	104.28 ± 31.72	97.7	NR	NR	85.83 ± 22.36	96 ± 89	NR	96.3	NR	NR	NR

Abbreviations: HT, hamstring tendon; LSI, limb symmetry index; Nm, Newton metres; NR, not reported; PT, patellar tendon; QT, quadriceps tendon.

In flexion, the QT consistently achieved the best results. At 6 months, both the QT and PT showed identical LSI values of 95.5 ± 12.8 at 90°/s. At 12 months, the QT demonstrated superior outcomes with LSI values of 102.9 ± 9.6 at 90°/s and 100.2 ± 14.9 at 180°/s. At 60°/s, the PT reached an LSI of 94, reported in a single study only [34].

By 24 months, the QT again achieved the highest outcomes, with LSI values of 111.88 ± 29.33 at 60°/s and 101.1 ± 5.1 at 90°/s. A complete table of all strength values is provided in the Supplementary Files (Table S3).

## DISCUSSION

The main finding of this systematic review is the emergence of distinct graft‐specific profiles of muscle strength recovery following ACLR. Analysis of 13 RCTs indicates that HT autografts are generally associated with more favourable recovery of knee extension strength, whereas QT autografts more consistently support superior flexion strength. PT autografts showed heterogeneous results and no consistent superiority. These observations underscore that graft selection is not a trivial decision, as it has measurable consequences for functional recovery and RTS.

The patterns observed are consistent with the principle of harvest site morbidity. Preservation of the extensor mechanism with HT autografts explains the superior recovery of quadriceps extension, while QT harvest directly compromises quadriceps integrity and may result in prolonged extensor weakness, although hamstring function is spared. Beyond absolute strength measures, persistent muscle activation deficits, particularly quadriceps inhibition, warrant closer attention [27]. Quadriceps deficits are more relevant than hamstring weakness, as they compromise dynamic stability, delay functional recovery, and are associated with an increased risk of posttraumatic osteoarthritis. Addressing these activation problems may therefore be as important as restoring peak torque, especially with regard to long‐term joint health. Quadriceps inhibition leads to a decrease in voluntary activation and maximal voluntary contraction, which is considered a prognostic factor for the development of osteoarthritis. The inhibition is caused by ACL injury and can be partially improved through physiotherapy or ACLR [3, 40]. Interestingly, studies have shown that quadriceps activation does not appear to return to the level observed in healthy individuals. Moreover, the activation deficit occurs bilaterally, which calls into question the validity of the LSI.

PT autografts, by affecting both extensor strength and anterior knee pain, show variable outcomes and may therefore not be recommended for quadriceps‐demanding or kneeling sports such as judo or weightlifting. PROMs only partially illustrate these differences: KOOS scores tended to favour HT, while Tegner and IKDC scores fluctuated, suggesting that PROMs depend on more than isolated strength measures. Although torque differences between grafts are statistically significant, their clinical impact remains uncertain, as absolute differences may not consistently translate into meaningful functional advantages.

A major limitation across studies is the lack of standardisation in strength assessment protocols. Variability in isokinetic protocols, use of gravitational correction and inconsistent application of the LSI complicate comparability. Although the LSI remains widely used as a benchmark for return‐to‐sport decisions, its validity has been questioned. Several studies have demonstrated that quadriceps strength is critical for successful RTS and for reducing reinjury risk [10, 15, 34, 35]. In line with this, RCTs and other reports have shown superior extension recovery with HT autografts but persistent quadriceps deficits with QT autografts, despite better hamstring recovery [7, 11, 13, 16, 24, 33]. At the same time, some authors questioned the predictive value of isokinetic measures and the LSI [39], while others emphasised that LSI often overestimates true recovery, increasing the risk of premature return‐to‐sport clearance [43]. These inconsistencies highlight the need for more reliable and clinically relevant neuromuscular assessment methods.

The clinical implications are considerable. No graft demonstrates universal superiority; rather, graft choice should be guided by patient‐specific requirements. Athletes dependent on strong extension may benefit more from HT autografts, while those requiring rapid hamstring flexion may be better served with QT autografts. Integrating graft‐specific recovery profiles into preoperative counselling can help align patient expectations with likely rehabilitation trajectories and long‐term outcomes.

Strengths of this review include a comprehensive multidatabase search, strict adherence to PRISMA guidelines and independent dual screening and data extraction, all of which reduce bias. Limitations must also be acknowledged: the restriction to studies published between 2014 and 2025 may have excluded relevant earlier data; heterogeneity in rehabilitation protocols, surgical techniques and outcome measures precluded meta‐analysis and limited generalisability; and most included studies reported follow‐up periods shorter than 2 years, leaving long‐term graft‐specific outcomes unclear. No sex‐specific subgroup analyses were performed, although both sexes were represented.

Future research should address whether sex influences graft‐specific recovery, as preliminary evidence suggests potential biomechanical differences between sexes in neuromuscular adaptation.

## CONCLUSIONS

The current evidence from RCTs suggests that distinct, graft‐specific patterns of muscle strength recovery occur following ACLR. HT tends to favour the recovery of extension strength, while QT supports better flexion strength recovery; PT outcomes remain inconsistent. Quadriceps strength remains a critical factor for a safe RTS and the prevention of reinjury. However, the widespread limitations in current assessment methodologies, including the conflicting evidence on the predictive value of the LSI, underscore the urgent need for more robust, standardised and clinically meaningful assessment tools. Future high‐quality RCTs with standardised protocols, larger patient cohorts and long‐term follow‐up are imperative to clarify the clinical implications of graft choice, validate objective RTS criteria and ultimately optimise rehabilitation strategies for all patients undergoing ACLR.

## AUTHOR CONTRIBUTIONS

Maximilian Heinz is the primary author of this paper. Maximilian Heinz and Yasemin Begüm Topkarci conducted the literature research to identify the current scientific evidence on graft differences in anterior cruciate ligament reconstruction. Maximilian Heinz and Yasemin Begüm Topkarci conducted the abstract and full‐text screening. Robert Prill monitored the conception of this study and provided necessary feedback for the study design. All authors have read and agreed to the published version of the manuscript. AI‐assisted editing was utilised for grammar and syntax optimisation using Grammarly Premium and DeepL Write. The visual abstract was generated with the assistance of Gemini Pro 3.0.

## CONFLICT OF INTEREST STATEMENT

The authors declare no conflicts of interest.

## ETHICS STATEMENT

Not applicable, as this study is a systematic review and does not involve human or animal participants.

## Supporting information

Table S1. Baseline characteristics of the included populations. two‐strand semitendinosus/gracilis (2ST/G); four‐strand semitendinosus (4ST); four‐strand semitendinosus/gracilis (4ST/G); anterior cruciate ligament (ACL); anterior cruciate ligament reconstruction (ACLR); Body mass index (BMI); Hamstring Tendon (HT); Limb Symmetry Index (LSI); Newton meters (Nm); not reported (NR); Patellar Tendon (PT); Quadriceps Tendon (QT); standard deviation (SD); semitendinosus/gracilis (ST/G).

Table S2. Overview of all PROMs. two‐strand semitendinosus/gracilis (2ST/G); four‐strand semitendinosus (4ST); four‐strand semitendinosus/gracilis (4ST/G); anterior cruciate ligament (ACL); Hamstring Tendon (HT); International Knee Documentation Committee (IKDC); Knee injury and Osteoarthritis Outcome Score (KOOS); not reported (NR); Patellar Tendon (PT); Quadriceps Tendon (QT); standard deviation (SD); semitendinosus/gracilis (ST/G).

Table S3. Overview of all strength outcomes. two‐strand semitendinosus/gracilis (2ST/G); four‐strand semitendinosus (4ST); four‐strand semitendinosus/gracilis (4ST/G); Hamstring Tendon (HT); Limb Symmetry Index (LSI); Newton meters (Nm); not reported (NR); Patellar Tendon (PT); Quadriceps Tendon (QT); standard deviation (SD); semitendinosus/gracilis (ST/G).

PRISMA_2020_checklist.

## Data Availability

All relevant data extracted and analysed during this review are fully presented within the article, including in the corresponding tables and figures.

## References

[1] Arida C , Tsikrikas CG , Mastrokalos DS , Panagopoulos A , Vlamis J , Triantafyllopoulos IK . Comparison of bone‐patella tendon‐bone and four‐strand hamstring tendon grafts for anterior cruciate ligament reconstruction: a prospective study. Cureus. 2021;13(10):e19197. 10.7759/cureus.19197 PMC8642134 34877191

[2] Barker TH , Stone JC , Sears K , Klugar M , Tufanaru C , Leonardi‐Bee J , et al. The revised JBI critical appraisal tool for the assessment of risk of bias for randomized controlled trials. JBI Evid Synth. 2023;21(3):494–506. 10.11124/JBIES-22-00430 36727247

[3] Chmielewski TL , Stackhouse S , Axe MJ , Snyder‐Mackler L . A prospective analysis of incidence and severity of quadriceps inhibition in a consecutive sample of 100 patients with complete acute anterior cruciate ligament rupture. J Orthop Res. 2004;22(5):925–930. 10.1016/j.orthres.2004.01.007 15304261

[4] Cristiani R , Mikkelsen C , Wange P , Olsson D , Stålman A , Engström B . Autograft type affects muscle strength and hop performance after ACL reconstruction: a randomised controlled trial comparing patellar tendon and hamstring tendon autografts with standard or accelerated rehabilitation. Knee Surg Sports Traumatol Arthrosc. 2021;29(9):3025–3036. 10.1007/s00167-020-06334-5 PMC8384829 33128587

[5] Duthon VB , Barea C , Abrassart S , Fasel JH , Fritschy D , Ménétrey J . Anatomy of the anterior cruciate ligament. Knee Surg Sports Traumatol Arthrosc. 2006;14(3):204–213. 10.1007/s00167-005-0679-9 16235056

[6] Ebert JR , Calvert ND , Radic R . A prospective randomized controlled trial investigating quadriceps versus hamstring tendon autograft in anterior cruciate ligament reconstruction. Am J Sports Med. 2024;52(3):660–669. 10.1177/03635465231222279 PMC10905979 38284303

[7] Girdwood M , Culvenor AG , Rio EK , Patterson BE , Haberfield M , Couch J , et al. Tale of quadriceps and hamstring muscle strength after ACL reconstruction: a systematic review with longitudinal and multivariate meta‐analysis. Br J Sports Med. 2025;59(6):423–434. 10.1136/bjsports-2023-107977 39389762

[8] Granan LP , Bahr R , Steindal K , Furnes O , Engebretsen L . Development of a national cruciate ligament surgery registry: the Norwegian National Knee Ligament Registry. Am J Sports Med. 2008;36(2):308–315. 10.1177/0363546507308939 17989167

[9] Griffin LY , Albohm MJ , Arendt EA , Bahr R , Beynnon BD , DeMaio M , et al. Understanding and preventing noncontact anterior cruciate ligament injuries: a review of the Hunt Valley II meeting, January 2005. Am J Sports Med. 2006;34(9):1512–1532. 10.1177/0363546506286866 16905673

[10] Grindem H , Snyder‐Mackler L , Moksnes H , Engebretsen L , Risberg MA . Simple decision rules can reduce reinjury risk by 84% after ACL reconstruction: the Delaware‐Oslo ACL cohort study. Br J Sports Med. 2016;50(13):804–808. 10.1136/bjsports-2016-096031 PMC4912389 27162233

[11] Herbawi F , Lozano‐Lozano M , Lopez‐Garzon M , Postigo‐Martin P , Ortiz‐Comino L , Martin‐Alguacil JL , et al. A systematic review and meta‐analysis of strength recovery measured by isokinetic dynamometer technology after anterior cruciate ligament reconstruction using quadriceps tendon autografts vs hamstring tendon autografts or patellar tendon autografts. Int J Environ Res Public Health. 2022;19(11):6764. 10.3390/ijerph19116764 PMC9180841 35682357

[12] Hewett TE , Di Stasi SL , Myer GD . Current concepts for injury prevention in athletes after anterior cruciate ligament reconstruction. Am J Sports Med. 2013;41(1):216–224. 10.1177/0363546512459638 PMC3592333 23041233

[13] Holmgren D , Noory S , Moström E , Grindem H , Stålman A , Wörner T . Weaker quadriceps muscle strength with a quadriceps tendon graft compared with a patellar or hamstring tendon graft at 7 months after anterior cruciate ligament reconstruction. Am J Sports Med. 2024;52(1):69–76. 10.1177/03635465231209442 PMC10762885 38164665

[14] Horstmann H , Petri M , Tegtbur U , Felmet G , Krettek C , Jagodzinski M . Quadriceps and hamstring tendon autografts in ACL reconstruction yield comparably good results in a prospective, randomized controlled trial. Arch Orthop Trauma Surg. 2022;142(2):281–289. 10.1007/s00402-021-03862-8 PMC8783919 33742222

[15] Hughes JD , Burnham JM , Hirsh A , Musahl V , Fu FH , Irrgang JJ , et al. Comparison of short‐term biodex results after anatomic anterior cruciate ligament reconstruction among 3 autografts. Orthop J Sports Med. 2019;7(5):2325967119847630. 10.1177/2325967119847630 PMC6545659 31211150

[16] Johnston PT , McClelland JA , Feller JA , Webster KE . Knee muscle strength after quadriceps tendon autograft anterior cruciate ligament reconstruction: systematic review and meta‐analysis. Knee Surg Sports Traumatol Arthrosc. 2021;29(9):2918–2933. 10.1007/s00167-020-06311-y 33026536

[17] Kaeding CC , Léger‐St‐Jean B , Magnussen RA . Epidemiology and diagnosis of anterior cruciate ligament injuries. Clin Sports Med. 2017;36(1):1–8. 10.1016/j.csm.2016.08.001 27871652

[18] Karimi‐Mobarakeh M , Mardani‐Kivi M , Mortazavi A , Saheb‐Ekhtiari K , Hashemi‐Motlagh K . Role of gracilis harvesting in four‐strand hamstring tendon anterior cruciate ligament reconstruction: a double‐blinded prospective randomized clinical trial. Knee Surg Sports Traumatol Arthrosc. 2015;23(4):1086–1091. 10.1007/s00167-014-2890-z 24531357

[19] Kouloumentas P , Kavroudakis E , Charalampidis E , Kavroudakis D , Triantafyllopoulos GK . Superior knee flexor strength at 2 years with all‐inside short‐graft anterior cruciate ligament reconstruction vs a conventional hamstring technique. Knee Surg Sports Traumatol Arthrosc. 2019;27(11):3592–3598. 10.1007/s00167-019-05456-9 30888448

[20] Lohmander LS , Englund PM , Dahl LL , Roos EM . The long‐term consequence of anterior cruciate ligament and meniscus injuries: osteoarthritis. Am J Sports Med. 2007;35(10):1756–1769. 10.1177/0363546507307396 17761605

[21] Mader K , Pennig D , Dargel J , Gotter M , Koebke J , Schmidt‐Wiethoff R . Biomechanics of the anterior cruciate ligament and implications for surgical reconstruction. Strategies Trauma Limb Reconstr. 2007;2(1):1–12. 10.1007/s11751-007-0016-6 PMC2321720 18427909

[22] Martin‐Alguacil JL , Arroyo‐Morales M , Martín‐Gomez JL , Monje‐Cabrera IM , Abellán‐Guillén JF , Esparza‐Ros F , et al. Strength recovery after anterior cruciate ligament reconstruction with quadriceps tendon versus hamstring tendon autografts in soccer players: a randomized controlled trial. Knee. 2018;25(4):704–714. 10.1016/j.knee.2018.03.011 29776815

[23] Mo IF , Harlem T , Faleide AGH , Strand T , Vindfeld S , Solheim E , et al. ACL reconstruction using quadrupled semitendinosus versus double‐stranded semitendinosus and gracilis autograft: 2‐year results from a prospective randomized controlled study. Am J Sports Med. 2024;52(8):1927–1936. 10.1177/03635465241254048 38845474

[24] Mouarbes D , Menetrey J , Marot V , Courtot L , Berard E , Cavaignac E . Anterior cruciate ligament reconstruction: a systematic review and meta‐analysis of outcomes for quadriceps tendon autograft versus bone–patellar tendon–bone and hamstring tendon autografts. Am J Sports Med. 2019;47(14):3531–3540. 10.1177/0363546518825340 30790526

[25] Ouzzani M , Hammady H , Fedorowicz Z , Elmagarmid A . Rayyan—a web and mobile app for systematic reviews. Syst Rev. 2016;5(1):210. 10.1186/s13643-016-0384-4 PMC5139140 27919275

[26] Page MJ , Moher D , Bossuyt PM , Boutron I , Hoffmann TC , Mulrow CD , et al. PRISMA 2020 explanation and elaboration: updated guidance and exemplars for reporting systematic reviews. BMJ. 2021;372:n160. 10.1136/bmj.n160 PMC8005925 33781993

[27] Pietrosimone B , Lepley AS , Kuenze C , Harkey MS , Hart JM , Blackburn JT , et al. Arthrogenic muscle inhibition following anterior cruciate ligament injury. J Sport Rehabil. 2022;31(6):694–706. 10.1123/jsr.2021-0128 35168201

[28] Popovic M , Myhre JR , Holen JIH , Gifstad T , Strand IL , Strand T , et al. Reduced knee flexion strength 18 years after ACL reconstruction with hamstring tendon versus patellar tendon. Am J Sports Med. 2024;52(11):2750–2757. 10.1177/03635465241271524 39221503

[29] Prill R , Karlsson J , Ayeni OR , Becker R . Author guidelines for conducting systematic reviews and meta‐analyses. Knee Surg Sports Traumatol Arthrosc. 2021;29(9):2739–2744. 10.1007/s00167-021-06631-7 34181055

[30] Prill R , Królikowska A , De Girolamo L , Becker R , Karlsson J . Checklists, risk of bias tools, and reporting guidelines for research in orthopedics, sports medicine, and rehabilitation. Knee Surg Sports Traumatol Arthrosc. 2023;31(8):3029–3033. 10.1007/s00167-023-07442-8 37145131

[31] Robson AWM . Ruptured crucial ligaments and their repair by operation. Br Med J. 1903;1(2208):1038–1041. PMC1431029 17861289

[32] Roger J , Bertani A , Vigouroux F , Mottier F , Gaillard R , Have L , et al. ACL reconstruction using a quadruple semitendinosus graft with cortical fixations gives suitable isokinetic and clinical outcomes after 2 years. Knee Surg Sports Traumatol Arthrosc. 2020;28(8):2468–2477. 10.1007/s00167-020-06121-2 32699919

[33] Runer A , Keeling L , Wagala N , Nugraha H , Özbek EA , Hughes JD , et al. Current trends in graft choice for primary anterior cruciate ligament reconstruction – part II: in‐vivo kinematics, patient reported outcomes, re‐rupture rates, strength recovery, return to sports and complications. J Exp Orthop. 2023;10(1):40. 10.1186/s40634-023-00601-3 PMC10073382 37014518

[34] Sasaki S , Tsuda E , Hiraga Y , Yamamoto Y , Maeda S , Sasaki E , et al. Prospective randomized study of objective and subjective clinical results between double‐bundle and single‐bundle anterior cruciate ligament reconstruction. Am J Sports Med. 2016;44(4):855–864. 10.1177/0363546515624471 26838934

[35] Schmitt LC , Paterno MV , Hewett TE . The impact of quadriceps femoris strength asymmetry on functional performance at return to sport following anterior cruciate ligament reconstruction. J Orthop Sports Phys Ther. 2012;42(9):750–759. 10.2519/jospt.2012.4194 PMC4157226 22813542

[36] Sinding KS , Nielsen TG , Hvid LG , Lind M , Dalgas U . Effects of autograft types on muscle strength and functional capacity in patients having anterior cruciate ligament reconstruction: a randomized controlled trial. Sports Med. 2020;50(7):1393–1403. 10.1007/s40279-020-01276-x 32125668

[37] Tang N , Eren M , Gurpinar T , Ozturkmen Y . A prospective randomized controlled study of hamstring and bone‐free quadriceps tendons autografts in arthroscopic ACL reconstruction. Eur J Orthop Surg Traumatol. 2024;34(1):293–301. 10.1007/s00590-023-03636-5 37468645

[38] Diermeier T , Rothrauff BB , Engebretsen L , Lynch AD , Ayeni OR , Paterno MV , et al. Treatment after anterior cruciate ligament injury: Panther Symposium ACL Treatment Consensus Group. Knee Surg Sports Traumatol Arthrosc. 2020;28(8):2390–2402. 10.1007/s00167-020-06012-6 PMC7524809 32388664

[39] Undheim MB , Cosgrave C , King E , Strike S , Marshall B , Falvey É , et al. Isokinetic muscle strength and readiness to return to sport following anterior cruciate ligament reconstruction: is there an association? A systematic review and a protocol recommendation. Br J Sports Med. 2015;49(20):1305–1310. 10.1136/bjsports-2014-093962 26105017

[40] Urbach D , Nebelung W , Becker R , Awiszus F . Effects of reconstruction of the anterior cruciate ligament on voluntary activation of quadriceps femoris: a prospective twitch interpolation study. J Bone Jt Surg Br. 2001;83(8):1104–1110. 10.1302/0301-620x.83b8.11618 11764420

[41] Van Grinsven S , Van Cingel REH , Holla CJM , Van Loon CJM . Evidence‐based rehabilitation following anterior cruciate ligament reconstruction. Knee Surg Sports Traumatol Arthrosc. 2010;18(8):1128–1144. 10.1007/s00167-009-1027-2 20069277

[42] Webster KE , Feller JA . Exploring the high reinjury rate in younger patients undergoing anterior cruciate ligament reconstruction. Am J Sports Med. 2016;44(11):2827–2832. 10.1177/0363546516651845 27390346

[43] Wellsandt E , Failla MJ , Snyder‐Mackler L . Limb symmetry indexes can overestimate knee function after anterior cruciate ligament injury. J Orthop Sports Phys Ther. 2017;47(5):334–338. 10.2519/jospt.2017.7285 PMC5483854 28355978

