# Supplementary material for: Hamstring autografts favour knee extension strength recovery while quadriceps autografts optimise flexion strength recovery: A systematic review of randomised controlled trials
Source: J Exp Orthop. 2026 Feb 18;13(1):e70665. doi: 10.1002/jeo2.70665 (PMC12914483; doi:10.1002/jeo2.70665)
Supplement: Supplementary file 1 — Table S1. Baseline characteristics of the included populations. two‐strand semitendinosus/gracilis (2ST/G); four‐strand semitendinosus (4ST); four‐strand semitendinosus/gracilis (4ST/G); anterior cruciate ligament (ACL); anterior cruciate ligament reconstruction (ACLR); Body mass index (BMI); Hamstring Tendon (HT); Limb Symmetry Index (LSI); Newton meters (Nm); not reported (NR); Patellar Tendon (PT); Quadriceps Tendon (QT); standard deviation (SD); semitendinosus/gracilis (ST/G). [file JEO2-13-e70665-s003.docx]

**Table S1 Baseline characteristics of the included populations.**

| **Author** | **Graft used** | **No. of patients** | **Age year ± SD** | **Age range** | **Sex male/female** | **BMI kg/m² ± SD** | **Adverse events** |
| --- | --- | --- | --- | --- | --- | --- | --- |
| **Arida et al. [1]** | BPTB | 30 | 29.93 ± 11.31 | NR | 23/7 | NR | ACL rerupture in the operated Knee (n=1) |
|  | HT (4ST/G) | 30 | 30.03 ± 11.7 | NR | 18/12 | NR | ACL rerupture in the operated Knee (n=6) |
| **Christiani et al. [4]** | BPTB (std.) | 40 | 29.3 ± 6.4 | NR | 25/15 | NR | ACLR contralateral (n=1) |
|  | HT (2ST/G std.) | 40 | 28 ± 6.3 | NR | 29/11 | NR | ACLR contralateral (n=1) |
|  | BPTB (Acc.) | 40 | 28.5 ± 5.5 | NR | 34/6 | NR | ACL rerupture in the operated Knee (n=3) |
|  | HT (2ST/G Acc.) | 40 | 28.8 ± 6.3 | NR | 27/13 | NR | ACL rerupture in the operated Knee (n=2) |
| **Ebert et al. [6]** | QT | 57 | 28.1 ± 8.2 | 16-47 | 28/29 | 26.6 ± 3.6 | ACL rerupture in the operated Knee (n=1), ACLR contralateral (n=1), Others (n=6) |
|  | HT (4ST) | 55 | 29.4 ± 7.7 | 16-47 | 28/27 | 26.3 ± 3.6 | ACLR contralateral (n=1), Other (n=5) |
| **Horstmann et al. [14]** | QT | 24 | 24.1 ± 3.6 | NR | 21/3 | 24.9 ± 3.8 | ACL rerupture in the operated Knee (n=3), Other (n=3) |
|  | HT (4ST/G) | 27 | 32.7 ± 11.4 | NR | 12/15 | 25.2 ± 4 | ACL rerupture in the operated Knee (n=1), Other (n=1) |
| **Karimi-Mobarakeh et al. [18]** | HT (4ST) | 58 | 29.9 ± 7.8 | NR | 48/10 | 23.4 ± 3.3 | ACLR contralateral (n=1) |
|  | HT (2ST/G) | 61 | 32.4 ± 6.3 | NR | 50/11 | 24.9 ± 4 | ACLR contralateral (n=2), Other (n=2) |
| **Kouloumentas et al. [19]** | HT (4ST) | 45 | 27.6 ± 11.4 | NR | 28/17 | 25.6 ± 2.8 | Other (n=1) |
|  | HT (4ST/G) | 45 | 29.7 ± 11 | NR | 27/18 | 24.8 ± 2.6 | Other (n=2) |
| **Martin-Alguacil et al. [22]** | QT | 26 | 18.7 ± 3 | NR | 23/3 | 23 ± 2.2 | ACL rerupture in the operated Knee (n=1) |
|  | HT | 25 | 19.2 ± 3.6 | NR | 16/9 | 23.5 ± 3.5 | ACL rerupture in the operated Knee (n=3) |
| **Mo et al. [23]** | HT (4ST) | 48 | 34.2 ± 9.5 | NR | 22/25 | 25 | ACL rerupture in the operated Knee (n=5), Other (n=19) |
|  | HT (4ST/G) | 49 | 34.1 ± 10.1 | NR | 20/29 | 24 | ACL rerupture in the operated Knee (n=2), Other (n=19) |
| **Popovic et al. [28]** | HT (4ST/G) | 49 | NR | 18-49 | 35/14 | NR | ACL rerupture in the operated Knee (n=4), ACLR contralateral (n=4), Others (n=15) |
|  | BPTB | 47 | NR | 18-49 | 28/19 | NR | ACL rerupture in the operated Knee (n=1), ACLR contralateral (n=4), Others (n=10) |
| **Roger et al. [31]** | HT (4ST) | 33 | 30.52 ± 8.9 | 19-48 | 26/7 | 24.3 ± 3.3 | ACL rerupture in the operated Knee (n=1) |
|  | HT (ST/G) | 27 | 30.3 ± 8.5 | 19-47 | 23/4 | 24.2 ± 2.4 | ACL rerupture in the operated Knee (n=1) |
| **Sasaki et al. [34]** | HT (4ST) | 67 | 28.2 ± 12.6 | NR | 32/25 | 23.2 ± 3.2 | ACL rerupture in the operated Knee (n=5), ACLR contralateral (n=4) |
|  | PT | 69 | 27 ± 11.9 | NR | 33/36 | 23.1 ± 3.1 | ACL rerupture in the operated Knee (n=3), ACLR contralateral (n=2) |
| **Sinding et al. [36]** | QT | 42 | 28.7 ± 6.4 | NR | 25/17 | 25.1 ± 3.2 | NR |
|  | HT (4ST/G) | 43 | 28.3 ± 6.2 | NR | 23/20 | 24.3 ± 3.2 | NR |
| **Tang et al. [37]** | QT | 17 | 28.06 ± 6.24 | NR | 17/0 | 25.1 ± 6.1 | Other (n=2) |
|  | HT (4ST/G) | 16 | 28.31 ± 8.55 | NR | 13/3 | 25.08 ± 4.7 | Other (n=5) |

two-strand semitendinosus/gracilis (2ST/G); four-strand semitendinosus (4ST); four-strand semitendinosus/gracilis (4ST/G); anterior cruciate ligament (ACL); anterior cruciate ligament reconstruction (ACLR); Body mass index (BMI); Hamstring Tendon (HT); Limb Symmetry Index (LSI); Newton meters (Nm); not reported (NR); Patellar Tendon (PT); Quadriceps Tendon (QT); standard deviation (SD); semitendinosus/gracilis (ST/G).
