# Supplementary material for: Hamstring autografts favour knee extension strength recovery while quadriceps autografts optimise flexion strength recovery: A systematic review of randomised controlled trials
Source: J Exp Orthop. 2026 Feb 18;13(1):e70665. doi: 10.1002/jeo2.70665 (PMC12914483; doi:10.1002/jeo2.70665)
Supplement: Supplementary file 2 — Table S2. Overview of all PROMs. two‐strand semitendinosus/gracilis (2ST/G); four‐strand semitendinosus (4ST); four‐strand semitendinosus/gracilis (4ST/G); anterior cruciate ligament (ACL); Hamstring Tendon (HT); International Knee Documentation Committee (IKDC); Knee injury and Osteoarthritis Outcome Score (KOOS); not reported (NR); Patellar Tendon (PT); Quadriceps Tendon (QT); standard deviation (SD); semitendinosus/gracilis (ST/G). [file JEO2-13-e70665-s001.docx]

**Table S2 Overview of all PROMs.**

| **Author** | **Graft used** | **No. of patients** | **Preoperative** | | | | **After 6 Months** | | | | **After 12 Months** | | | | **After 24 Months** | | | |  |
| --- | --- | --- | --- | --- | --- | --- | --- | --- | --- | --- | --- | --- | --- | --- | --- | --- | --- | --- | --- |
|  |  |  | **Tegner ± SD** | **IKDC ± SD** | **Lysholm ± SD** | **KOOS ± SD** | **Tegner ± SD** | **IKDC ± SD** | **Lysholm ± SD** | **KOOS ± SD** | **Tegner ± SD** | **IKDC ± SD** | **Lysholm ± SD** | **KOOS ± SD** | **Tegner ± SD** | **IKDC ± SD** | **Lysholm ± SD** | **KOOS ± SD** |  |
| **Arida et al. [1]** | BPTB | 30 | NR | NR | NR | NR | 6.14 ± 1.33 | 83.28 ± 10.45 | 88.79 ± 9.31 | NR | 7.97 ± 1.43 | 89.71 ± 10.48 | 95.34 ± 6.31 | NR | NR | NR | NR | NR |  |
|  | HT (4ST/G) | 30 | NR | NR | NR | NR | 5.64 ± 1.25 | 79.64 ± 12.26 | 87.64 ± 6.73 | NR | 7.24 ± 1.64 | 86.88 ± 11.59 | 93.68 ± 4.71 | NR | NR | NR | NR | NR |  |
| **Ebert et al. [6]** | QT | 57 | 3.6 ± 1.7 | 49.7 ± 17.9 | 58.9 ± 23.2 | 56.3 ± 17.4 | 4.8 ± 1.7 | 71.6 ± 14.8 | 81.2 ± 12 | 69 ± 8.6 | 6 ± 1.7 | 82 ± 14.2 | 88.3 ± 11.1 | 73.8 ± 6.8 | NR | NR | NR | NR |  |
|  | HT (4ST) | 55 | 3.2 ± 1.7 | 50.6 ± 18.9 | 63.8 ± 21.3 | 60.1 ± 19.9 | 4.7 ± 1.2 | 74.5 ± 12.5 | 85 ± 11.5 | 72.5 ± 5.9 | 6.3 ± 1.8 | 86.1 ± 10.8 | 89.9 ± 9.1 | 76.4 ± 5.6 | NR | NR | NR | NR |  |
| **Horstmann et al. [14]** | QT | 24 | NR | 66.8 ± 16.9 | 72.3 ± 13.2 | NR | NR | NR | NR | NR | NR | NR | NR | NR | NR | 89.3 ± 12.2 | 90.4 ±11.9 | NR |  |
|  | HT (4ST/G) | 27 | NR | 59.0 ± 17.2 | 60.4 ± 18.5 | NR | NR | NR | NR | NR | NR | NR | NR | NR | NR | 83.7 ± 12.7 | 83.5 ±17.4 | NR |  |
| **Karimi-Mobarakeh et al. [18]** | HT (4ST) | 58 | NR | 62.58 ±9.54 | 55.1 ± 8.9 | NR | NR | NR | NR | NR | NR | 83.5 ± 6.3 | 86.2 ± 4.6 | NR | NR | NR | NR | NR |  |
|  | HT (2ST/G) | 61 | NR | 61.4 ± 8.64 | 57.4 ± 9.3 | NR | NR | NR | NR | NR | NR | 80.8 ± 6.8 | 85.3 ± 4.9 | NR | NR | NR | NR | NR |  |
| **Kouloumentas et al. [19]** | HT (4ST) | 45 | NR | 41.9 ± 12.7 | 45.6 ± 15.7 | 68.6 ± 6.6 | NR | NR | NR | NR | NR | NR | NR | NR | NR | 83.6 ± 8.2 | 97.7 ± 2.1 | 95.3 ± 3.8 |  |
|  | HT (4ST/G) | 45 | NR | 43.6 ± 14 | 44.8 ± 17.5 | 65.9 ± 7.2 | NR | NR | NR | NR | NR | NR | NR | NR | NR | 78.5 ± 9.9 | 96.6 ±2.2 | 95.8 ± 3.6 |  |
| **Martin-Alguacil et al. [22]** | QT | 26 | 7.6 | NR | NR | NR | 8.93 | NR | NR | NR | 9.38 | NR | NR | NR | 9.38 | NR | NR | NR |  |
|  | HT | 25 | 7.58 | NR | NR | NR | 8.88 | NR | NR | NR | 9.59 | NR | NR | NR | 9.27 | NR | NR | NR |  |
| **Mo et al. [23]** | HT (4ST) | 48 | NR | 55.9 ± 14.8 | NR | NR | NR | NR | NR | NR | NR | NR | NR | NR | NR | 80.5 ± 14.4 | NR | NR |  |
|  | HT (4ST/G) | 49 | NR | 54.4 ± 12.6 | NR | NR | NR | NR | NR | NR | NR | NR | NR | NR | NR | 79.2 ± 14.6 | NR | NR |  |
| **M. Popovic et al. [28]** | HT (4ST/G) | 49 | 4 | NR | 67 | NR | NR | NR | NR | NR | 7 | NR | 93 | NR | 7 | NR | 93 | NR |  |
|  | BPTB | 47 | 4 | NR | 65 | NR | NR | NR | NR | NR | 6 | NR | 92 | NR | 6 | NR | 92 | NR |  |
| **Roger et al. [32]** | HT (4ST) | 33 | NR | 57.6 ± 14.7 | NR | NR | NR | NR | NR | NR | NR | NR | NR | NR | NR | 80.2 ± 12.5 | NR | NR |  |
|  | HT (ST/G) | 27 | NR | 59.8 ± 13.2 | NR | NR | NR | NR | NR | NR | NR | NR | NR | NR | NR | 83.6 ± 13.6 | NR | NR |  |
| **Sasaki et al. [34]** | HT (2x 2ST) | 67 | 6.2 ± 2.0 | NR | NR | NR | NR | NR | NR | NR | NR | NR | NR | NR | 6.5 ± 1.7 | NR | NR | NR |  |
|  | PT | 69 | 6.7 ± 1.6 | NR | NR | NR | NR | NR | NR | NR | NR | NR | NR | NR | 4.8 ± 2.2 | NR | NR | NR |  |
| **Sinding et al. [36]** | QT | 42 | NR | 56 ± 18 | NR | NR | NR | NR | NR | NR | NR | 76 ± 17 | NR | NR | NR | NR | NR | NR |  |
|  | HT (4ST/G) | 43 | NR | 60 ± 14 | NR | NR | NR | NR | NR | NR | NR | 76 ± 15 | NR | NR | NR | NR | NR | NR |  |
| **Tang et al. [37]** | QT | 17 | NR | 66.88 ± 8.09 | 67.94 ± 11.16 | 71.47 ± 11.57 | NR | NR | NR | NR | NR | NR | NR | NR | NR | 87.12 ± 8.09 | 90.00 ± 4.26 | 85.59 ± 5.05 |  |
|  | HT (4ST/G) | 16 | NR | 63.66 ± 9.84 | 65.25 ± 11.17 | 68.49 ± 10.98 | NR | NR | NR | NR | NR | NR | NR | NR | NR | 90.13 ± 6.99 | 90.06 ± 7.33 | 92.41 ± 3.08 |  |
|  |  |  |  |  |  |  |  |  |  |  |  |  |  |  |  |  |  |  |  |
|  |  |  |  |  |  |  |  |  |  |  |  |  |  |  |  |  |  |  |  |
|  |  |  |  |  |  |  |  |  |  |  |  |  |  |  |  |  |  |  |  |
|  |  |  |  |  |  |  |  |  |  |  |  |  |  |  |  |  |  |  |  |
|  |  |  |  |  |  |  |  |  |  |  |  |  |  |  |  |  |  |  |  |
|  |  |  |  |  |  |  |  |  |  |  |  |  |  |  |  |  |  |  |  |
|  |  |  |  |  |  |  |  |  |  |  |  |  |  |  |  |  |  |  |  |
|  |  |  |  |  |  |  |  |  |  |  |  |  |  |  |  |  |  |  |  |
|  |  |  |  |  |  |  |  |  |  |  |  |  |  |  |  |  |  |  |  |
|  |  |  |  |  |  |  |  |  |  |  |  |  |  |  |  |  |  |  |  |

two-strand semitendinosus/gracilis (2ST/G); four-strand semitendinosus (4ST); four-strand semitendinosus/gracilis (4ST/G); anterior cruciate ligament (ACL); Hamstring Tendon (HT); International Knee Documentation Committee (IKDC); Knee injury and Osteoarthritis Outcome Score (KOOS); not reported (NR); Patellar Tendon (PT); Quadriceps Tendon (QT); standard deviation (SD); semitendinosus/gracilis (ST/G).
