# Supplementary material for: Hamstring autografts favour knee extension strength recovery while quadriceps autografts optimise flexion strength recovery: A systematic review of randomised controlled trials
Source: J Exp Orthop. 2026 Feb 18;13(1):e70665. doi: 10.1002/jeo2.70665 (PMC12914483; doi:10.1002/jeo2.70665)
Supplement: Supplementary file 3 — Table S3. Overview of all strength outcomes. two‐strand semitendinosus/gracilis (2ST/G); four‐strand semitendinosus (4ST); four‐strand semitendinosus/gracilis (4ST/G); Hamstring Tendon (HT); Limb Symmetry Index (LSI); Newton meters (Nm); not reported (NR); Patellar Tendon (PT); Quadriceps Tendon (QT); standard deviation (SD); semitendinosus/gracilis (ST/G). [file JEO2-13-e70665-s004.docx]

**Table S3 Overview of all strength outcomes.**

| **Author** | **Graft used** | **No. of patients** | **Device used** | **LSI ± SD after 6 Months** | | | | | | **LSI ± SD after 12 Months** | | | | | | **LSI ± SD after 24 Months ± SD** | | | | | |
| --- | --- | --- | --- | --- | --- | --- | --- | --- | --- | --- | --- | --- | --- | --- | --- | --- | --- | --- | --- | --- | --- |
|  |  |  |  | **Extension** | | | **Flexion** | | | **Extension** | | | **Flexion** | | | **Extension** | | | **Flexion** | | |
|  |  |  |  | **60 °/s** | **90 °/s** | **180 °/s** | **60 °/s** | **90 °/s** | **180 °/s** | **60 °/s** | **90 °/s** | **180 °/s** | **60 °/s** | **90 °/s** | **180 °/s** | **60 °/s** | **90 °/s** | **180 °/s** | **60 °/s** | **90 °/s** | **180 °/s** |
| **Christiani et al. [4]** | BPTB (std.) | 40 | Biodex System 3 | NR | 71.4 | NR | NR | 95.5 | NR | NR | 85.8 | NR | NR | 97.7 | NR | NR | 92.6 | NR | NR | 96.3 | NR |
|  | HT (2ST/G std.) | 40 | Biodex System 3 | NR | 81.6 | NR | NR | 85.4 | NR | NR | 90.5 | NR | NR | 86 | NR | NR | 91.2 | NR | NR | 87.1 | NR |
|  | BPTB (acc.) | 40 | Biodex System 3 | NR | 72.2 | NR | NR | 98.2 | NR | NR | 85.1 | NR | NR | 100.8 | NR | NR | 92.9 | NR | NR | 97.8 | NR |
|  | HT (2ST/G acc.) | 40 | Biodex System 3 | NR | 82.9 | NR | NR | 88.1 | NR | NR | 92.8 | NR | NR | 89.5 | NR | NR | 93.5 | NR | NR | 91.6 | NR |
| **Ebert et al. [6]** | QT | 57 | Isosport International | NR | 60.9 ± 14.8 | NR | NR | 95.5 ± 12.8 | NR | NR | 77.3 ± 15.9 | NR | NR | 102.9 ± 9.6 | NR | NR | 89.3 ± 10.9 | NR | NR | 101.1 ± 5.1 | NR |
|  | HT (4ST) | 55 | Isosport International | NR | 71.2 ± 16.8 | NR | NR | 87.7 ± 12.1 | NR | NR | 85.3 ± 13.1 | NR | NR | 93.8 ± 11 | NR | NR | 92.5 ± 8.2 | NR | NR | 94 ± 8 | NR |
| **Kouloumentas et al. [19]** | HT (4ST) | 45 | Biodex System 4 Pro | NR | NR | NR | NR | NR | NR | NR | NR | NR | NR | NR | NR | 90 ± 10 | NR | 90 ± 10 | 90 ± 10 | NR | 100 ± 10 |
|  | HT (4ST/G) | 45 | Biodex System 4 Pro | NR | NR | NR | NR | NR | NR | NR | NR | NR | NR | NR | NR | 90 ± 10 | NR | 90 ± 10 | 90 ± 10 | NR | 90 ± 10 |
| **Mo et al. [23]** | HT (4ST) | 48 | Biodex System 3 | NR | NR | NR | NR | NR | NR | NR | NR | NR | NR | NR | NR | 87.4 ± 16.5 | NR | NR | 88.7 ± 21.3 | NR | NR |
|  | HT (4ST/G) | 49 | Biodex System 3 | NR | NR | NR | NR | NR | NR | NR | NR | NR | NR | NR | NR | 88 ± 10.4 | NR | NR | 86 ±18.6 | NR | NR |
| **Roger et al. [32]** | HT (4ST) | 33 | Biodex System 3 | 62 ± 16 | NR | NR | 75 ± 13 | NR | NR | NR | NR | NR | NR | NR | NR | 86 ± 12 | NR | NR | 83 ± 16 | NR | NR |
|  | HT (ST/G) | 27 | Biodex System 3 | 63 ± 17 | NR | NR | 73 ± 11 | NR | NR | NR | NR | NR | NR | NR | NR | 81 ± 17 | NR | NR | 86 ± 11 | NR | NR |
| **Sasaki et al. [34]** | HT (2 X 2ST) | 67 | Cybex 6000 | 76 ± 16 | NR | NR | 88 | NR | NR | 86 | NR | NR | 96 | NR | NR | 91 ± 12 | NR | NR | 91 ± 15 | NR | NR |
|  | PT | 69 | Cybex 6000 | 69 ± 18 | NR | NR | 92 | NR | NR | 84 | NR | NR | 94 | NR | NR | 89 ±13 | NR | NR | 96 ± 89 | NR | NR |
| **Sinding et al. [36]** | QT | 42 | Humac Norm CSMI | NR | NR | NR | NR | NR | NR | 84.1 ± 12.7 | NR | 83.4 ± 13.4 | 92.3 ± 19.2 | NR | 100.2 ± 14.9 | NR | NR | NR | NR | NR | NR |
|  | HT (4ST/G) | 43 | Humac Norm CSMI | NR | NR | NR | NR | NR | NR | 90.2 ± 11.2 | NR | 88.1 ± 10.2 | 85.7 ± 17 | NR | 83.4 ± 17.2 | NR | NR | NR | NR | NR | NR |
| **Tang et al. [37]** | QT | 17 | Humac Norm CSMI | NR | NR | NR | NR | NR | NR | NR | NR | NR | NR | NR | NR | 119.76 ± 41.99 | NR | NR | 111.88 ± 29.33 | NR | NR |
|  | HT (4ST/G) | 16 | Humac Norm CSMI | NR | NR | NR | NR | NR | NR | NR | NR | NR | NR | NR | NR | 145.06 ± 37.37 | NR | NR | 105.75 ± 22.89 | NR | NR |
| **Author** | **Graft used** | **No. of patients** | **Device used** | **Newton-metre ± SD after 6 Months ± SD** | | | | | | **Newton-metre ± SD after 12 Months** | | | | | | **Newton-metre ± SD 24 Months** | | | | | |
|  |  |  |  | **Extension** | | | **Flexion** | | | **Extension** | | | **Flexion** | | | **Extension** | | | **Flexion** | | |
|  |  |  |  | **60 °/s** | **90 °/s** | **180 °/s** | **60 °/s** | **90 °/s** | **180 °/s** | **60 °/s** | **90 °/s** | **180 °/s** | **60 °/s** | **90 °/s** | **180 °/s** | **60 °/s** | **90 °/s** | **180 °/s** | **60 °/s** | **90 °/s** | **180 °/s** |
| **Arida et al. [1]** | BPTB | 30 | Cybex 6000 human version 2004 | 125.59 ± 59 | NR | 93.07 ± 38.4 | 98.34 ± 32.07 | NR | 77.86 ± 26.37 | 153.17 ± 50.84 | NR | 109.9 ± 33.52 | 104.28 ± 31.72 | NR | 85.83 ± 22.36 | NR | NR | NR | NR | NR | NR |
|  | HT (4ST/G) | 30 | Cybex 6000 human version 2004 | 123.38 ± 43.49 | NR | 85.04 ± 30.56 | 79.29 ± 23.07 | NR | 63.13 ± 20.95 | 138.08 ± 48.91 | NR | 99.79 ± 30.32 | 86.17 ± 27.84 | NR | 71.38 ± 24.64 | NR | NR | NR | NR | NR | NR |
| **Karimi-Mobarakeh et al. [18]** | HT (4ST) | 58 | MicroFet2 | NR | NR | NR | NR | NR | NR | NR | 71.8 ± 10.5 | NR | NR | 68.3 ± 9.1 | NR | NR | NR | NR | NR | NR | NR |
|  | HT (2ST/G) | 61 | MicroFet2 | NR | NR | NR | NR | NR | NR | NR | 73.7 ± 11.8 | NR | NR | 65.5 ± 8.6 | NR | NR | NR | NR | NR | NR | NR |
| **Martin-Alguacil et al. [22]** | QT | 26 | Genu 3 | 125.7 ± 40.3 | NR | 104 ± 29.1 | 136.4 ± 39.8 | NR | 77.86 ± 26.37 | 139.5 ± 47.3 | NR | 115.6 ± 33.2 | 147.4 ± 37.8 | NR | 127.4 ± 34.1 | NR | NR | NR | NR | NR | NR |
|  | HT | 25 | Genu 3 | 139.9 ± 47.9 | NR | 113.6 ± 32.3 | 116.5 ± 41.3 | NR | 109.7 ± 34.2 | 144.94 ± 51.4 | NR | 114.7 ± 32.9 | 131.3 ± 40.4 | NR | 112.2 ± 29.3 | NR | NR | NR | NR | NR | NR |
| **Popovic et al. [28]** | HT (4ST/G) | 49 | Biodex | NR | NR | NR | NR | NR | NR | NR | NR | NR | NR | NR | NR | NR | NR | NR | NR | NR | NR |
|  | BPTB | 47 | Biodex | NR | NR | NR | NR | NR | NR | NR | NR | NR | NR | NR | NR | NR | NR | NR | NR | NR | NR |
| **Athor** | **Graft used** | **No. of patients** | **Device used** | **Injured-Healthy in Nm ± SD after 6 months** | | | | | | **Injured-Healthy in Nm ± SD after 12 months** | | | | | | **Injured-Healthy in Nm ± SD after 12 months** | | | | | |
|  |  |  |  | **Extension** | | | **Flexion** | | | **Extension** | | | **Flexion** | | | **Extension** | | | **Flexion** | | |
|  |  |  |  | **60 °/s** | **90 °/s** | **180 °/s** | **60 °/s** | **90 °/s** | **180 °/s** | **60 °/s** | **90 °/s** | **180 °/s** | **60 °/s** | **90 °/s** | **180 °/s** | **60 °/s** | **90 °/s** | **180 °/s** | **60 °/s** | **90 °/s** | **180 °/s** |
| **Horstmann et al. [14]** | QT | 24 | CON-TREX Multi-Joint | -1 ± 1 | NR | NR | -0.2 ± 0.8 | NR | NR | -0.6 ± 0.7 | NR | NR | 0 ± 0.7 | NR | NR | -0.3 ± 1.2 | NR | NR | -0.1 ± 1 | NR | NR |
|  | HT (4ST/G) | 27 | CON-TREX Multi-Joint | -1 ± 1.1 | NR | NR | -0.7 ± 1 | NR | NR | -0.4 ± 0.7 | NR | NR | -0.4 ± 0.9 | NR | NR | -0.2 ± 1.2 | NR | NR | -0.5 ± 0.8 | NR | NR |

two-strand semitendinosus/gracilis (2ST/G); four-strand semitendinosus (4ST); four-strand semitendinosus/gracilis (4ST/G); Hamstring Tendon (HT); Limb Symmetry Index (LSI); Newton meters (Nm); not reported (NR); Patellar Tendon (PT); Quadriceps Tendon (QT); standard deviation (SD); semitendinosus/gracilis (ST/G).
